# Ultrasonic Processing of Food Waste to Generate Value-Added Products

**DOI:** 10.3390/foods11142035

**Published:** 2022-07-09

**Authors:** Yue Wu, Shunyu Yao, Bhakti Anand Narale, Akalya Shanmugam, Srinivas Mettu, Muthupandian Ashokkumar

**Affiliations:** 1 Sonochemistry Group, School of Chemistry, The University of Melbourne, Parkville, VIC 3010, Australia; wuyw7@student.unimelb.edu.au (Y.W.); shunyuy3@student.unimelb.edu.au (S.Y.); 2 Food Processing Business Incubation Centre, National Institute of Food Technology, Entrepreneurship and Management-Thanjavur, Thanjavur 613005, India; bhakti@iifpt.edu.in; 3 Centre of Excellence in Non-Thermal Processing, National Institute of Food Technology, Entrepreneurship and Management-Thanjavur, Thanjavur 613005, India; 4 Chemical and Environmental Engineering, School of Engineering, RMIT University, Melbourne, VIC 3000, Australia

**Keywords:** ultrasound processing, food waste, extraction, bio-energy products, sustainable goals

## Abstract

Ultrasonic processing has a great potential to transform waste from the food and agriculture industry into value-added products. In this review article, we discuss the use of ultrasound for the valorisation of food and agricultural waste. Ultrasonic processing is considered a green technology as compared to the conventional chemical extraction/processing methods. The influence of ultrasound pre-treatment on the soluble chemical oxygen demand (SCOD), particle size, and cell wall content of food waste is first discussed. The use of ultrasonic processing to produce/extract bioactives such as oil, polyphenolic, polysaccharides, fatty acids, organic acids, protein, lipids, and enzymes is highlighted. Moreover, ultrasonic processing in bioenergy production from food waste such as green methane, hydrogen, biodiesel, and ethanol through anaerobic digestion is also reviewed. The conversion of waste oils into biofuels with the use of ultrasound is presented. The latest developments and future prospective on the use of ultrasound in developing energy-efficient methods to convert food and agricultural waste into value-added products are summarised.

## 1. Introduction

Food waste consists of amino acids, proteins, lipids, polysaccharide, fibre, and other compounds [1]. Generally, it is generated by fresh produce markets, the agricultural industry, commercial restaurants and canteens, food processing plants, and food supply chain industries. Nowadays, large amounts of food waste are generated, and the Food and Agriculture Organization has reported that over 1 billion tons of food from human consumption are wasted [2]. Therefore, food loss and food waste directly affect the economy, environment, and society, which may ultimately hinder the development and progress achieved through efficient agriculture production. In fact, around 931 million tons of edible food have been wasted at downstream stages, in which household consumption accounted for 61%, followed by food service (26%) and retail (13%) [3]. Usually, food waste is treated by landfilling, incineration, composting, and anaerobic digestion [4]. However, the organic matter content in food waste can reach up to 80% of the dry mass [5]. In addition, food waste has a high water content and abundant organic matter. These characteristics, which can be used to upgrade the food waste to value-added products, are wasted in the treatment of food waste using incineration, landfill, or anaerobic digestion technologies [6]. For example, anaerobic digestion has some limitations, such as long operation time, low efficiency, and environmental pollution. Similarly, landfills require enough space and a long degradation time to be able to treat food waste. Moreover, incineration is associated with high cost and energy consumption (fuel and oxygen). Therefore, novel technologies are emerging to improve the utilization of food waste in order to produce value-added products.

**Figure 1:**
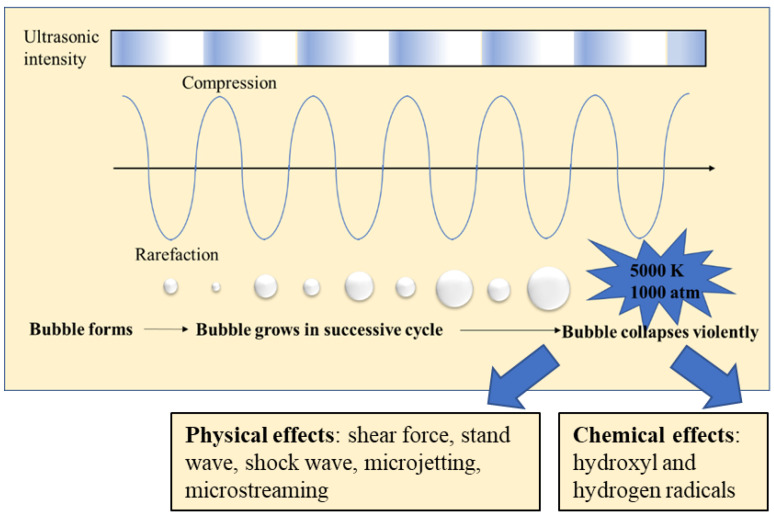
A schematic representation of the acoustic cavitation phenomenon.

The chemical treatment of food waste to extract valuable compounds is not sustainable as it uses petrochemical-based solvents. For the sustainable valorisation of food waste, there is a need to develop clean technologies that do not use chemicals derived from fossil fuel. Ultrasound is one viable option emerging as a clean technique that can be utilised in food processing areas, for extraction [7,8,9], homogenisation [10], and physicochemical property modification [11]. When power ultrasound (20 kHz frequency) is applied to the materials, the cavitation bubbles are generated in a liquid medium. The cavitation bubbles oscillate and collapse, generating high shear forces (Figure 1). The tremendous energy is released during the cavitation [12], which then leads to increased mass transfer and cell disruption for extraction purposes [13,14]. Although high-intensity ultrasound is an effective method for cell rupture, specific kinds of spores and microorganisms [15] sometimes have hard cell walls that ultrasound cannot fully disrupt as a standalone method. Currently, ultrasound is a mature technology that can be utilised independently or associated with other technology. With other technique combinations, ultrasound can thus be highly effective. General ultrasonic combinations serve as a pre-treatment of food waste for enhanced bioenergy production, including physical, thermal [16], microwave [17,18,19], high hydrostatic pressure [7], and chemical approaches, which are used together with enzymes [20,21], ionic liquid [22], acid [23,24], and alkali [25,26]. For the purpose of creating improved properties, ultrasound can act as a modification method, directly influencing the physicochemical characteristics of food waste materials. Therefore, ultrasound can be utilized to generate valuable compounds from food waste.

This review focuses on the application of ultrasound and its effects on food waste. First, the influence of ultrasound pre-treatment on soluble chemical oxygen demand, particle size, and the cell wall content of food waste is presented. Then, the ultrasound-assisted extraction of oil, polyphenol, polysaccharides, and proteins from various food waste is summarised. Next, the ultrasound-promoted production of bioenergy, including bio-methane, bio-hydrogen, biodiesel, and bioethanol from food waste is discussed. Then, we discuss the current challenges and future developments in relation to the utilization of ultrasound on food waste. 

## 2. The Influence of Ultrasound Pre-Treatment on Food Waste Materials

### 2.1. The Impact of Ultrasound on Organic Substance Solubilisation

Pre-treatment is a crucial step prior to enhanced bio-energy production. Anaerobic digestion yields can be accelerated using pre-treatment. The typical conversion process of food waste to bio-energy products includes sequential complex biochemical reactions during anaerobic digestion, which significantly impacts biogas production yield. It has been reported that organic substrate availability and mass transfer are the significant parameters that affect anaerobic digestion [27]. It is acknowledged that improving the organic matter solubilisation can effectively enhance the conversion rate, thus increasing the bio-energy production yields [28,29,30].

**Figure 2:**
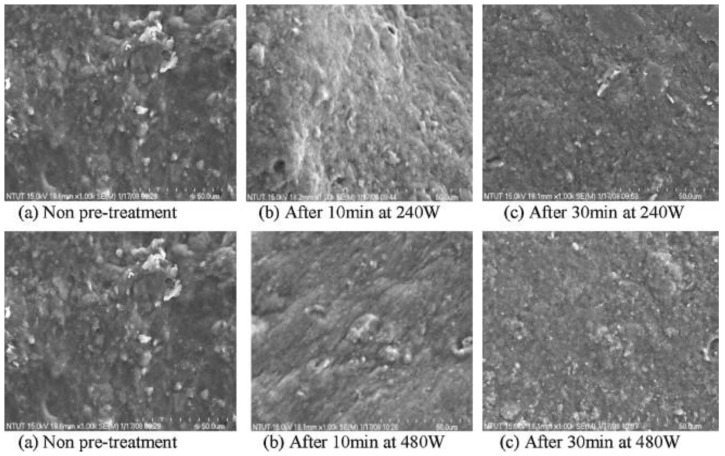
The morphology of the floc structure of sludge before and after ultrasonic pre-treatment under SEM [36]. Note: different lowercases represents food waste without ultrasound treatment (**a**), with sonication for 10 min (**b**) and with sonication for 30 min (**c**).

Ultrasound is an effective way to disrupt floc and cells due to the extreme pressures, high shear forces, violent turbulence, primary oxidative radicals, among other physical effects generated by acoustic cavitation [31,32,33]. Nevertheless, the efficiency of sonication was based on other parameters, such as ultrasound power, ultrasound time, and raw materials [34,35]. Figure 2 shows the morphology of the floc structure of the sludge before and after sonication pre-treatment using SEM. The sludge floc has an overall uneven structure at the start, and while sonication proceeds, a more consistent structure and smaller size can be observed, owing to floc disintegration [36]. The morphology shown in the figure indicates that with a longer sonication time, higher sonication power had more influence on the floc disintegration process.

The chemical oxygen demand (COD) is normally used to determine the number of organic substances in the waste [37], and soluble chemical oxygen demand (SCOD) can be an indicative method of reflecting the influence of ultrasound on organic substance solubilisation, which has been listed in Table 1. It can be observed in the table that different parameters can not only affect the sonication efficiency, as mentioned, but they can also exert their influence on organic substance solubilisation. Abhijit [38] reported an increase of ~56% in SCOD release as compared to samples without sonication. Wang et al. [39] investigated the influence of sonication on food waste in relation to the production of volatile fatty acids. They first applied the same ultrasound conditions onto various total solid (TS) content food waste. Higher ΔSCOD was observed in lower TS content samples due to the intensified power density in the system. Joshi and Gogate [40] carried out a systematic study of different US parameters of food waste in order to improve the anaerobic digestion process for biogas production. The highest SCOD value of 18,500 ± 20 mg/L (with a 61.5% increase) was observed for the following conditions: 10 min sonication time, 0.4 W/mL power density, and 60% duty cycle (US pulse mode), which was treated as the optimum. Generally, a longer sonication time and higher power will give a higher SCOD value [41,42,43,44]. However, a decrease in SCOD value was observed when the US power density increased due to a higher power density, leading to the degradation of organic compounds and the cushioning effect [45]. In addition to the standalone utilisation of sonication, Elbeshbishy et al. [46] investigated the use of ultrasound with other techniques and their combined effects on organic matter solubilisation. They combined the thermal (heat) and chemical (acid and base) method with ultrasound, and the highest SCOD increase of 33% was obtained for the Ultrasound-base pre-treatment.

### 2.2. The Influence of Ultrasound on Particle Size

**Figure 3:**
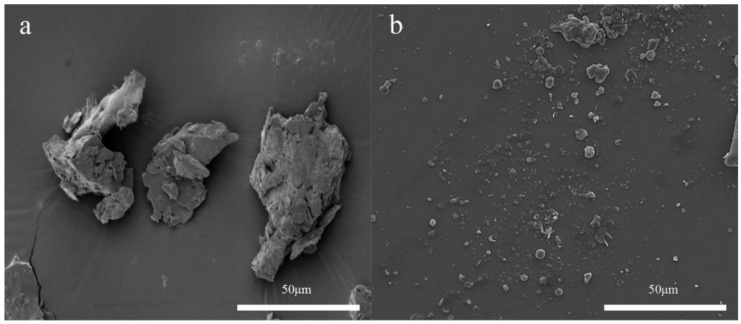
The morphology of food waste samples under SEM: (**a**) control (untreated) food waste sample, (**b**) sonicated food waste sample using a 35 mm horn (at 0.34 W/mL power). The SEM pictures were obtained at a magnification of 2000 [70]. Note: The scale bar is 50 μm.

As mentioned earlier, sonication is a practical approach for cell wall disruption [65,66,67]. The cell rupture occurs due to high shear forces and the generation of the extreme pressures and high local temperature conditions through acoustic cavitation. These conditions bring about characteristic modification via particle size alteration [68,69,70]. Therefore, ultrasound has been extensively applied in the biochemical processing area, such as in wastewater treatment [71], drug delivery [72], enhanced protein functionality [73], surface cleaning [74], among others. Today, ultrasound is also used in the pre-treatment of food waste to make it more viable for ensuing bio-energy production. Usually, the assistance of sonication can lead to particle size reduction, as depicted in Figure 3, hence expanding the surface area for the enzymatic attack [70]. Pejin et al. [75] implied that the ultrasound pre-treatment of triticale can reduce particle size, thereby enhancing the saccharification yield. Likewise, Nitayavardhana et al. [76] utilised ultrasound at 8 W/mL power density for 40 s to rupture the lignocellulosic cell structure in the cassava chip slurries; accordingly, particle size was considerably decreased from 600 to 15 μm. Similarly, another case of a significant reduction in particle size was reported by Khanal et al. [77]; the particle size of corn declined by nearly 20-fold with the application of ultrasonic pre-treatment under a high-power mode. An integrated investigation of the effectiveness of ultrasonic pre-treatment of food waste was carried out by Li et al. [70], who suggested that particle size reduction is a function of power and time of sonication. For instance, the particle size of the samples treated with 0.39 W/mL and 0.34 W/mL ultrasound was found to have decreased by 74% and 34%, respectively, when compared to the control sample. In terms of the waste mixture, the study of the sonication pre-treatment of a mixture of food waste and cardboard performed by Begum et al. [48] showed that ultrasound still had a beneficial effect on droplet size reduction, which decreased 10-fold for a 1:1 mix rate with ultrasonic pre-treatment as compared to the untreated samples.

### 2.3. The Effects of Ultrasound on Typical Polysaccharides

The composition of food waste varies between different species. Land plants (vegetables, fruits) are the most representative type of food waste (Table 2). The cell wall of these materials is ruptured as a result of ultrasound pre-treatment. Their fundamental components such as typical polymers (cutin, pectin) and a majority of polysaccharides (lignin, cellulose, hemicellulose) may be affected [78]. In other food wastes, the polysaccharides are primarily composed of lignin, cellulose, and hemicellulose, with lignin governing the interaction net between each content, thus leading to a complicated and obstinate structure [40]. This complex structure, typically with high lignocellulosic material content, makes further production less efficient. However, it is conceivable that after ultrasonic pre-treatment, these available polysaccharides are readily decomposed into monomeric sugars and further used for bio-energy production [79]. Moreover, it has been established that ultrasound is a proven predominant approach for lignin barrier disruption, hemicellulose removal, and cellulose crystallinity reduction. The removal of the lignin content is ascribed to bond oxidation during the ultrasound [80], and hemicellulose removal is attributed to its amorphous nature [81]. The reduction in lignin and hemicellulose content leads to an improvement in cellulose content [60]. Therefore, enhancing the accessibility to cellulose (disintegration of hydrogen bond in cellulose microfibrils) allows for further bio-energy production yield to be improved [42]. In previously reported studies, Ji et al. [82] investigated how different ultrasonic parameters affect these polysaccharides in vegetable wastes. It was discovered that in the single frequency sonication, the lignin removal rate decreased from 85.66 to 78.36%, when ultrasound frequency increased from 20 to 60 kHz, which might be ascribed to the decrease in ultrasonic cavitation. Perrone et. al. [83] noted that sugarcane bagasse samples pre-treated with sonication can effectively reduce hemicellulose and lignin content but enhance cellulose content from 41.3 to 51.8%. Likewise, Velmurugan and Muthukumar [84] also reported a dramatic reduction in hemicellulose and lignin percentage with alkaline-ultrasound pre-treatment, but a significant increase in the cellulose content of sugarcane bagasse was observed. On the contrary, high losses of cellulose content in spent coffee waste were reported by Ravindran [85], which might be attributed to a prolonged exposure to the ultrasonic environment. Comparably, the research carried out by Zhang et al. [86] found similar results in rice hull, which indicated that extrusion pre-treatment could lead to the degradation of cellulose, thereby reducing cellulose content even with the use of sonication. The delignification of biomass to hydrolyse lignin is a critical step in preparing the biomass for a further step in downstream processing such as fermentation [87]. Saratale et al. obtained a lignin removal rate of 25% in wheat waste with an individual ultrasonic pre-treatment [88]. Ong et al. [89] reported a delignification rate of 36.4% in oil palm fronds with an ultrasound-assisted deep eutectic solvent (DES) pre-treatment. However, Gaudino et al. [90] achieved the highest delignification rate of 45% in wheat straw, owing to the fact that higher lignin depolymerization was triggered by greater ultrasonic power. Mohapatra et al. [91] conducted a systematic study of the delignification efficiency on the Pennisetum sp. They reported that the highest delignification rates of 80.4% and 82.1% for Denanath grass (DG) and Hybrid Napier grass (HNG) were obtained with ultrasound-assisted pre-treatment. Furthermore, they also recognized that a more significant fraction of the sonication duty cycle, a higher temperature, and more intensive ultrasonic power have beneficial impacts on lignin elimination. 

**Figure 4:**
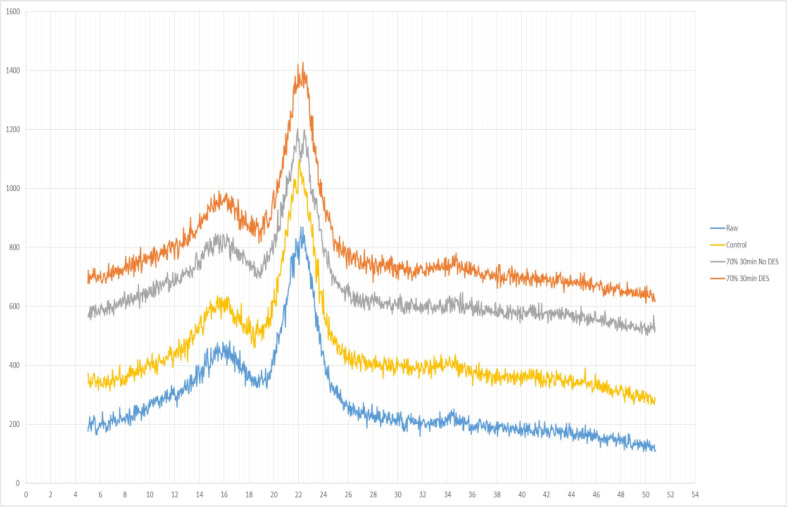
The X-ray diffraction (XRD) spectra of control and pre-treated samples [89].

In addition to the polysaccharide content changes, the crystallinity of the amorphous component may also be altered after pre-treatment. The impacts of ultrasound are generated mainly by ultrasonic cavitation, which leads to violent shear forces, intense turbulence, high temperature, and pressure in a localized area [94]; this can in turn lead to the disruption of lignin content, thus increasing the accessibility of the cellulose component. Moreover, the increase in the crystallinity index after sonication is ascribed to the higher cellulose content, and the results can also be used to determine the extent of the delignification process [45]. Figure 4 demonstrates representative XRD patterns of the control and the pre-treated samples; XRD was utilized to examine the intensity of amorphous and crystalline areas at 18° and 22° (2θ), respectively [95]. It can be observed from the figure that at the 2θ = 22° crystalline region, a higher crystallinity was achieved after ultrasonic pre-treatment. The higher index is due to ultrasonic pre-treatment disrupting the lignin network and increasing the available surface area, as previously mentioned. Likewise, Velmurugan and Muthukumar [96] reported that in sugarcane bagasse, the crystallinity index of the pre-treated ultrasonic sample was found to be 16% more than the control sample. Sindhu et al. [97] observed that the crystallinity index in chilli post-harvest residue for the control and ultrasonic pre-treated samples were 35.43% and 52.32%, respectively. They also achieved the highest crystallinity index of 63.62% with the sono-assisted acid pre-treatment. Nonetheless, some investigations have also indicated that ultrasonic pre-treatment might decrease the crystallinity index. Hydroxyl groups in cellulose macromolecules form intramolecular and intermolecular hydrogen bonds, resulting in a variety of structured crystal lattices [98]. As mentioned, the extreme conditions caused by ultrasound can effectively disintegrate hydrogen bonds in the cellulose component and modify its crystalline arrangement, thus leading to the crystallinity reduction [99]. Jin et al. [100] investigated sweet potato and reported that ultrasound damaged its cellulose structure, thus resulting in a reduction in its crystallinity zone (2θ = 22°). Similarly, Ninomiya et al. [101] discovered that ultrasonic pre-treatment in bamboo powder was more effective in crystalline reduction than in the thermal method. It was found that sample products from the ultrasound treatment could be further reduced by 6.1% more than the thermal treatment.

## 3. Ultrasound-Assisted Extraction of Bioactives from Food Waste

### 3.1. Conventional Methods of Extraction

**Figure 5:**
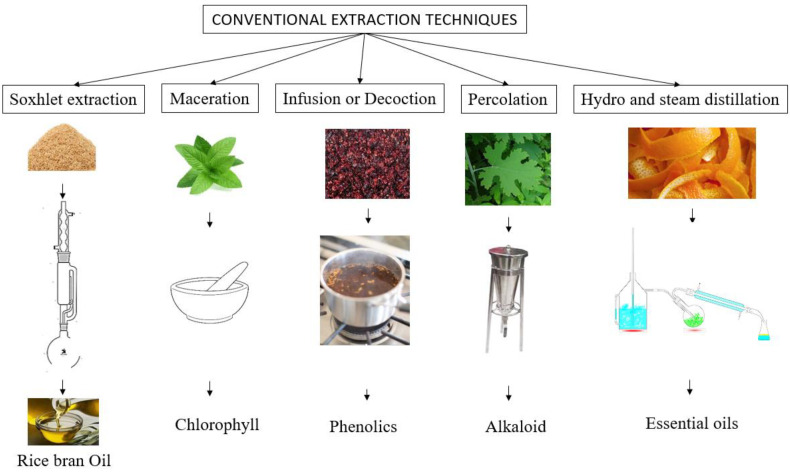
Schematic illustration of various conventional methods of extraction of bioactives or useful materials from different food wastes.

Extraction is a mass-transport process where a solute migrates from a matrix to a solvent [102]. Distillation by water, distillation by steam, distillation by a combination of water and steam, Soxhlet extraction, maceration, decoction, and percolation are the most common conventional processes used in the extraction of useful materials from waste [103]. Liquid hot water saccharification (particularly optimization at the lab scale) and steam explosion (especially suited for industry) are generally practiced amongst all the thermal treatments of extraction. Both treatments use a similar matrix destructuring mechanism, which is dependent on biomass treatment using hot water at high-pressure and its release, either slowly (in a liquid hot-water treatment) or abruptly (steam explosion) [104]. Recent research has discovered that a steam explosion-assisted extraction is highly effective, particularly for the extraction of phenolic acid, and the recovery yield also increases with a rise in residence time and temperature [105]. Conventional treatments are economical; however, there are multiple factors that bring out the complexity in the process, especially with the use of solvents, the longer processes, and the sequences in the processing method. The density, viscosity, reactivity, the volume of utilization of the medium employed in the process are some of the factors that complicate the processing. Even with the challenges above, Soxhlet extraction is very much used as a base technique for comparison against any new processes [106]. Modern tools of extraction include the use of ultrasound [107], pulse electric field [108], supercritical extraction and microwave [109], etc. Ultrasound is considered as one major emerging tool for the extraction of useful materials from food waste [107]. Ultrasound-based extraction has the following advantages in comparison to all emerging technologies: higher yield, lesser medium of extraction, lower capital, lesser time and energy, low operating temperature, and prevention of purity [107]. Figure 5 represents the schematic illustration of various conventional methods of extraction of bioactives or useful materials from different food wastes.

### 3.2. Extraction Using Ultrasound

The use of ultrasound in extraction processes has grown significantly in recent decades, especially in the food and natural product fields, for both traditional and environmentally novel extraction approaches. Ultrasound-assisted extraction (UAE) is an environmentally friendly, low-cost method that adheres to all green chemistry standards. When compared to conventional extraction processes, UAE reduces the extraction time, energy, and solvent consumption, while maintaining the high purity of the end product [110]. Shear forces, pressure changes, agitation, cavitation, microjets, radical generation as well as fragmentation, erosion, capillarity, and sonoporation all contribute to the extraction impact of ultrasound, which occurs both in the solvent and in the matrix, as pointed out by Chemat et al. [110]. The cavitation effects of ultrasound appear to be the main cause of this sonication effect. Microbubbles are formed when ultrasonic vibrations create a series of low-pressure and high-pressure zones in a medium such as solvent. Because of the rapid and continual pressure changes, these bubbles grow and eventually burst. When the bubbles implosively collapse, breaking points emerge on the surface of the cell, thus contributing to the increased transfer of mass in and out of cells. This approach is valuable for the extraction of useful materials such as polyphenols and many different molecules from a range of food wastes that employ different solvents [111].

### 3.3. Ultrasonic Extraction of Useful Materials from Cereal Processing Waste

With the significant production of husk, bran, and germ as the principal cereal by-products, the large yields are followed by post-harvest losses in all phases of product conversion. Plant by-products are prone to microbial breakdown due to their high water content and significant organic load, resulting in environmental difficulties when discarded and increasing waste treatment expenses for food makers. The recovery of biogenic substances from low-cost plant by-products could provide economic and environmental benefits as well as a cash return for enterprises rather than incurring disposal expenditures [112]. Food by-products such as wheat and rice brans, oat hulls, and wheat germ contain significant levels of phytosterols and polysaccharides. Phytosterols have been shown to have a positive impact on human health. Bioactive polysaccharides found mostly in cereals are known as β-glucans [113]. In comparison to rye (1–2%) and wheat (1%), barley (3–11%) and oat (3–7%) had higher levels of β-glucans. 

Ultrasonication is a technique for destructing plant cell walls and releasing polysaccharides and other difficult-to-extract substances at lower temperatures and in less time. Small intervals of UAE were utilized for separating components of hemicellulose (mostly heteroxylans that are rich in polyphenols) from commercial wheat bran that gave sugar yields comparable to regular alkaline extraction. Furthermore, the procedure could be cut in half by using less sodium hydroxide, resulting in a 60% reduction in time [114]. UAE allows for a reduction in traditional solvent use or, alternatively, the use of water or greener solvents such as ethanol; hence, it is a reliable green technology. In one study, the response surface approach was used to optimise a UAE technique for the extraction of major phenolic acids (ferulic, salicylic, 2-hydroxycinnamic, p-coumaric, t-ferulic, caffeic, and vanillic acids) from the bran layers of certain key ancient wheat species. Of all the phenolic acids studied, ethanol had the highest extraction yield for any solvent type [115]. Another study predicted and optimised the UAE for monomeric anthocyanin and phenolic chemicals from black and purple rice bran. Extraction yield was studied in relation to pH, temperature, solvent concentration, and extraction time. Purple rice bran extracts had a greater total phenolic content of 2232 mg Gallic acid equivalent (GAE)/100 g and higher monomeric anthocyanin content of 34.86 mg C3G/L as compared to black rice bran extracts, which had values of 31.95 mg C3G/L and 1978.76 mg GAE/100 g under the optimum conditions. Furthermore, for black (753.89 mg GAE/100 g and 18.75 mg C3G/L) and purple rice bran (778.98 mg GAE/100 g and 21.82 mg C3G/L), the anthocyanin and total phenolic content yields using UAE were significantly higher than the usual extraction procedure [116]. Likewise, from corn cob waste, xylan was separated using UAE, with a reduction in the time of extraction, the amount of alkali usage, and the extraction temperature. In comparison with the xylan extracted without ultrasonic treatment in a 5% solution of NaOH, the composition of xylan and the physical structure remained unaltered but with improved bioactivity [114]. Moreover, polysaccharides extracted from the stem of sijiaoling with the use of UAE also showed better antioxidant capacity in comparison to the extraction process using hot water [117]. Kumar et al. [107] explored ultrasound in the production of oil from rice bran. Similarly, the oil from wheat germ was extracted by Teslic et al. [118] with a yield of 10.67%.

### 3.4. Ultrasonic Extraction of Useful Materials from Fruit and Vegetable Processing Waste

Fruit processing waste consists primarily of 40 to 50% cellulose, 10 to 25% lignin, and 20 to 30% hemicellulose and other polysaccharides [112]. Similarly, the wastes are also rich in polyphenol, pectins, and dietary fibre concentrations as compared to the edible components. The most common phenolic chemicals found in fruit and vegetable wastes are phenolic acids, flavonoids, and tannins [119]. Several research works have shown that using UAE increases pectin, a polysaccharide output, and reduces extraction time. Peels of pomegranate, orange, grapefruit, eggplant, grape pomace, and tomato waste have all resulted in pectin yields of above 25% in the UAE [107]. Wang et al. [120] examined the chemical and UAE extraction of pectin from grapefruit peel and found that UAE produced a greater yield (16.34%) and that the time of extraction was reduced by 37.78%. Likewise, de Oliveira et al. [121] found a 1.6-fold rise in the productivity of pectin on the UAE of peels obtained from passion fruit when compared to chemical extraction. Another study of UAE improved the extraction yield of pectin to 53% in the case of mango peel phenolic residue and to 31% in the case of rehydrated mango peel [122]. A substantial association between pectin emergence and tissue swelling was reported by Xu et al. [123], who came to the conclusion that the vegetal tissue disruption using ultrasonic treatment is the major mechanism for improving the extractability by UAE. Similarly, crude polysaccharide was successfully extracted from rambutan fruit peel using an efficient UAE approach. The ultrasonic process parameters of 110 W, 53 °C, and 41 min showed the best result. This could be used in any value addition of food products [124]. A different study on the dual frequency ultrasound treatment of ginger leaves and stem (DFGLS) and the triple frequency ultrasound treatment of ginger leaves and stem (TPGLS) for the extraction of polysaccharides resulted in a yield of 9.74% and 10.50%, respectively. The TFGLS polysaccharides had a larger total sugar, uronic acid, and sulphate radical concentrations than the DFGLS polysaccharides, but a lower molecular weight. The antioxidant capabilities of TFGLS polysaccharides were higher for scavenging DPPH, 2,2′-azinobis (3-ethylbenzothiazoline-6-sulphonic acid) (ABTS), superoxide radicals, and hydroxyl; they also had a high reducing power and chelating activity as well as better emulsifying property, hygroscopicity, and foamability. These findings suggest that dual- and triple-frequency ultrasound can enhance the functional properties and biological activities of polysaccharides obtained from leaves and stems of ginger [125]. A higher production of cavitation bubbles and the resulting physical phenomena in the case of multi-frequency ultrasound produces the above effects noted in TFGLS polysaccharides [126]. The polysaccharides obtained from various food wastes are used in the formulation of value-added food products such as texturizing, stabilizing, thickening, and emulsifying additives [127]. Likewise, the soluble dietary fibres are known to reduce cholesterol and the associated risk of cardiovascular diseases [128]. The findings of Zhang et al. [129] imply that papaya peel is a good source of soluble dietary fibre (SDF), and that ultrasound-assisted alkaline extraction yielded 36.99% of SDF. When comparing the SDF of the alkaline extraction method to the SDF of the ultrasound-assisted method (u-SDF), the u-SDF contains more essential amino acids and minerals. Furthermore, u-SDF has shown improvements in thermal stability and water holding, oil holding, and swelling capacity, implying that this process could significantly improve the properties of SDF, which could be a viable component in functional product formulations. Furthermore, the monosaccharide present is predominantly pectic saccharide, which belongs to the low-methoxy pectin family and forms stable food-grade gels with calcium ions [129]. Few studies have made a comparison between conventional solvent extraction and UAE solvent extraction for dietary fibres; those that did found that the UAE of the dietary fibre from citrus changshan-huyou peels [130] and apple pomace [131] gave higher yields, took less process time, and used lower temperatures. Similarly, UAE dietary fibre from culinary banana bract, peels of papaya, and residues of soyabean had superior yield, thermal stability, purity, water-holding, swelling, and oil-holding capabilities than alkaline-extracted dietary fibre [129,132]. Another comparative study between the UAE and the Soxhlet method on oil obtained from seeds of Moringa peregrina was carried out by Mohammadpour et al. [133]. The greatest oil yield with UAE was 53.101%, whereas it was 43% with the Soxhlet process after 11 h of extraction. The peroxide value (PV) of oil extracted by the Soxhlet method was higher than the UAE method, while other chemical properties such as iodine value, antioxidant activity (DPPH percent), and total phenolic content of oil extracted by the Soxhlet method were lower than those obtained by the UAE method. As a result of several physiochemical investigations, it has been discovered that the UAE approach increases oil quality. Overall, it can be concluded that the UAE can be a cost-effective and economically viable technology. Some researchers have combined ultrasound with other processing tools such as microwave, pulse electric field to extract useful materials. In one such study [134], pectin was extracted from fig skin by ultrasound/microwave-aided extraction (UMAE). A combined sonication process with a time of 21.35 min, microwave power at 580.9 W, and an irradiation time of 11.67 min was found to provide the optimum conditions for the extraction yield (11.71%). The yield was higher for UAE and the hot water extraction method, which were 8.74% and 6.95%, respectively. A microstructure evaluation of the UMAE-treated sample revealed that there is enough disintegration of plant material, resulting in the increased release of pectic substance. The pectin obtained was studied for various bioactivities such as anti-radical, anti-oxidant, and anticancer activities. An improvement in functional property was observed due to uronic acids and many sugars such as glucose, fucose, arabinose, galactose, rhamnose, and mannose, with an average molecular weight of 6.89×103 kDa. Hence, this pectin from fig skin is a novel ingredient which could enable the formation of healthier function foods [134].

### 3.5. Ultrasonic Extraction of Useful Materials from Oilseeds and Nuts, Pulses and Legume Processing Waste

UAE technology was used to obtain the sunflower by-product pectin (SFBP). The UAE factors were successfully tuned, and the best result was 11.15% (irradiation period 30 min, temperature 33 °C, and ultrasound power 400 W). SFBP was found to have a low-esterified galacturonic acid content (72.94%). It was observed to have long side branches of galactan, arabinogalactan, and arabinan (with an average molecular weight of 175 kDa) under these sonication conditions. SFBP appeared to have strong thermal stability according to the thermal analysis. The results of functional qualities such as solubility, water-holding capacity, oil-holding capacity, emulsifying capacity, emulsion stability, foam capacity, foam stability, and antioxidant capabilities revealed that SFBP had a better value than commercial ones [135]. Similarly, UAE with parameters such as sonication time at 10 min, ultrasound power at 200 W, and a liquid-to-solvent ratio of 1.5 yielded 12.78% of pectin from walnut processing waste. In these conditions, the resultant pectin was rich in galacturonic acid (69.44%) and high in degree of esterification (59.21%), as determined by the NMR and FTIR spectra. Furthermore, the molecular weight distribution analysis revealed that the pectin obtained had low molecular weight (6.30–158.48 kDa) and was heterogeneous in nature. The walnut pectin XRD spectrum revealed an amorphous structure with few crystalline regions. Walnut pectin also demonstrated good water and oil retention capacities, radical scavenging activity, and emulsifying characteristics [136].

The goal of another study was to see how UAE affected the yield of polysaccharide gums (PSG) from flaxseed meal. Anti-nutritional components such as cyanide (HCN) and tannins were dramatically reduced with the use of ultrasound-assisted extraction. When the extraction temperature (°C) and amplitude level (%) were changed, and all other variables were kept constant, the PSG yield extracted from partially defatted flaxseed meal samples were in the range of 7.24–11.04%. Accompanied by chemical analysis, the viscosity, solution stability, and the foaming and emulsifying capabilities were investigated to see if they could be used as a new food additives [137]. In a different study, UAE was shown to be a superior approach for getting larger yields of proteases (up to 330 IU) and α-amylases (825 IU) while reducing extraction time and obtaining a more concentrated product [138]. Likewise, Chanioti and Tzia [139] studied the goodness of UAE in oil recovery from olive pomaces. The yield of oil extraction and its unsaponifiable matter, total phenol content, and antioxidant activity were all assessed. The extraction yield was found to be 2.5–4.4% of unsaponifiable matter, 10.9% oil, 0.16–0.21 mg Trolox g-1 oil, and 0.14–0.26 mg GA g-1 oil for the total phenolic content. Hence, the emerging extraction technology of ultrasound in combination with inexpensive and natural raw materials such as olive pomace confirmed it to be an economical alternative to conventional methods of extraction, in accordance with the demands of the food industry and sustainable development. A recent work developed and validated an ultrasound-assisted extraction technique for extracting phenolic compounds from Moroccan almond cold-pressed oil residue. The ideal extraction conditions were determined using response surface methods, which included using an ultrasound frequency of 27.0 kHz and 53.0% (*v*/*v*) aqueous ethanol as a green solvent for a 29.4 min extraction time. Upon comparison with the conventional heat reflux extraction, the ultrasound-assisted extraction allowed for a significant rise in extraction efficiency. Under ideal conditions for ultrasound-assisted extraction, total phenolic content was found to be 13.86 mg/g dry weight. The main phenolic chemicals found in the valuable waste were identified by doing an HPLC analysis, which used chlorogenic acid, protocatechuic acid, followed by p-hydroxybenzoic acid, and p-coumaric acid [140].

Another study optimised canola oil extraction with the help of 35 kHz, 800 W ultrasonic waves using solvents such as hexane-isopropanol and hexane at the ratio of 2:3. With the help of the Box–Behnken design, the effects of the ultrasound treatment time, solvent-to-canola (sample) ratio, and extraction temperature on the oxidative stability and yield of extracted oil were investigated. The optimum conditions obtained for canola oil extraction with an efficiency of 22.39% using hexane were: ultrasonic treatment for 87 min at 55 °C and a solvent-to-canola proportion of 6.39 (% *v*/*w*), according to the data. When compared to Soxhlet extraction, the results showed that ultrasound waves greatly improved extraction efficiency. When a hexane–isopropanol combination was used, the extraction efficiency increased. An increase in ultrasonic treatment resulted in a significant improvement in oxidative stability. In comparison to the extracted oil using hexane as a solvent, the oxidative stability of the extracted oil with a mixed solvent (hexane–isopropanol) was higher. The fatty acid compositions of the ultrasound-assisted and the Soxhlet-extracted oils were not significantly different when compared using gas chromatography [141]. In a combination process, an ultrasound-assisted aqueous enzymatic extraction method was employed in the cooking pre-treatment to extract date seed oil. The cooking temperature and solvent ratio can affect the oil quality because a prolonged heating process will cause the thermal degradation of oil, resulting in the formation of hydroperoxides and other products resulting from oil degradation and oxidation [142]. The results showed that a sample-to-solvent ratio of 1: 2 and a cooking period of 40 min were the best conditions for extracting date seed oil. Overall, the findings of this study show that it was critical to choose the right sample-to-solvent ratio and to use a cooking pre-treatment in order to extract oil with high yield and quality. This study found that by using the cooking pre-treatment, the innovative, ultrasound-assisted, aqueous enzymatic extraction procedure improved the extraction of oil from the sample [142].

In a recent study, soluble dietary fibres (SDFs) were extracted from black soybean hulls using ultrasound–microwave co-modification and enzyme modification (cellulase and hemicellulase). SDF structural, physical, and chemical characteristics as well as their binding capacity with cholesterol were investigated previous to the modification and also after the modification. SDFs obtained using the ultrasound–microwave co-modified hulls and the enzyme-modified hulls had a molecular weight reduction of 33.21% and 45.29%, respectively, from the raw black soybean hulls. The extracted SDFs modified by the ultrasound–microwave method had a water-holding capacity of 3.79 g/g, a water-swelling capacity of 1.39 mL/g, and an oil-holding capacity of 1.14 g/g, and an increase of 9.54%, 23.01%, and 17.53%, respectively, over the raw SDF values. When compared to raw SDFs, the enzyme-modified SDFs had a water-holding capacity of 3.59 g/g, a water-selling capacity of 1.25 mL/g, and an oil-holding capacity of 1.03 g/g, signifying an increase of 3.76%, 10.62%, and 6.19%, respectively. On comparing the values of the cholesterol-binding capacity of SDFs modified by the ultrasound–microwave and enzyme methods to raw SDFs, the values were 13.82 and 12.34 mg/g, respectively, indicating a 47.98% and a 32.20% increase [143]. This can help in lowering cholesterol and in the improvement of cardiovascular health. Overall, the UAE process helps to further improve the extraction of useful components such as oil, polysaccharides, phenolic compounds, dietary fibres, pectins, etc., through the imposed physical effects of acoustic cavitation and the implied mass transfer. A combination with other techniques has always proved to be beneficial, as confirmed by the above discussion.

### 3.6. Ultrasonic Extraction Design and Optimization Methods

Ultrasonic extraction is a multi-variable system that depends on power, frequency of ultrasound, sonication time as well the viscosity of the medium being sonicated. The design of experiments (DOE) as well as the optimization of experimental parameters are crucial for improving the extraction and energy efficiency of sonication systems. In this regard, several ultrasonic extraction design and optimization methods have been explored [144,145,146]. Typical optimization methods are the Response Surface Methodology (including the Box–Behnken design), the Plackett–Burman design, and the orthogonal experimental design. Typically, for high-intensity ultrasonic extraction, frequency is fixed at around 20 kHz. Then, the optimization variables left are the power of ultrasound, sonication time, and the viscosity of the medium.

The viscosity of the medium is generally dependent on the ratio of raw materials to water. The higher the concentration of raw materials, the higher the viscosity is. Recently, we have shown that the efficient extraction of lipids by the rupture of microalgae cells required around 20% solids [14]. However, such an optimization needs to be carried out for individual systems since the viscosity dependence on solid concentration is material-dependent. Additionally, the rheological behaviour of the medium needs to be characterized. The rheological behaviour of solid slurry could be Newtonian, shear-thinning, or shear-thickening. The shear-thinning materials would perform better when ultrasonicated as compared to the shear-thickening material. The decrease in viscosity with shear (with applied ultrasound) would aid in the extraction, whereas the increase in viscosity for the shear-thickening material would hinder the extraction due to energy dissipation and an insufficient penetration of ultrasonic waves.

One of the well-used process optimization methods is the RSM (Response Surface Methodology) [146]. RSM optimises the process of selecting the factorial variable so that the desired response from the combination of variables is either maximized or minimized. The RSM can be used to screen for factorial variables first, followed by a three-level factorial response study. The response contour plots as a function of the system variables are used to locate the variable combination, either for a minimized or a maximized response. In a process system involving multiple parameters and their interaction, the use of RSM reduces the number of experimental trials needed to optimise the system, hence making the optimization less laborious and saving costs.

Generally, the first-order polynomial response (*Y*) as a function of independent system variables (*n*) is obtained, as shown below from the Plackett–Burman design method [147]:

(1) $$Y=\beta_{o}+\sum_{i=1}^{n}\beta_{i}X_{i}$$

where *Y* is the predicted response, (*β_o_*) is the intercept, *β_i_* is the linear regression coefficient, and *X_i_* is the coded independent variable. This model assumes no interactions between the independent variables. The PB design method is used for the screening of important independent variables that can have a significant effect on the response.

Once the screening has been completed, three important independent variables are chosen, to which the Box–Behnken Design is applied, as shown below:

(2) $$Y=\beta_{o}+\sum_{i=1}^{3}\beta_{i}X_{i}+\sum_{i=1}^{3}\beta_{ii}X_{i}^{2}+\sum_{i=1}^{2}\sum_{j=i+1}^{3}\beta_{ij}X_{i}X_{j}$$

where *Y* is the predicted response; (*β_o_*) is the intercept; *β_i_* is the linear, *β_ii_* is the quadratic, and *β_ij_* is the interaction regression coefficient; *X_i_* and *X_j_* are the coded independent variables. Generally, the Design-Expert Software (Stat-Ease Inc., Minneapolis, MN, USA) was used for the regression analysis of the experimental design, in data analysis, and therefore to obtain the quadratic polynomial response function.

### 3.7. Alterations after Exposure to Ultrasound and the Possible Effects and Consequences for Product Quality

Solvents are typically used for plant extraction in the food industry, which is polluting, labour-intensive, and expensive. Soxhlet extraction is generally used with the most popular oil extraction method. The benefits of employing low-frequency ultrasound have been reported, which shows a rise in the recovery of the compounds under softer extraction conditions within a shorter period of time, with the help of more environmentally friendly organic solvents such as ethanol or by using other green solvents. Although extraction yields are higher when compared to conventional extraction procedures, food items with high fat content exposed to ultrasonic-for-extraction reasons have also shown alterations in the organoleptic and/or chemical properties [148].

The samples had different fatty acid compositions, and the presence of the lipid-degrading compounds (2E, 4E)-deca-2,4-dienal and limonene (Z)-hept-2-enal indicated that the kiwi seed oil had undergone oxidation. This oil has a high amount of polyunsaturated fatty acids (PUFAs), 57% of which are linolenic acid (C18:3), and a low amount of tocols, i.e., tocopherol/tocotrienol (35 mg kg^−1^), which can affect the stability it has against oxidation [149]. However, the value of peroxides rose during the lipid extraction of flaxseed with ultrasonic assistance, which led the scientists to speculate that free radicals may possibly have been produced [150]. Despite the two soybean varieties improving the yield of oil with the UAE (11.2%), a general drop in unsaturated fatty acids and a rise in saturated ones, at a 3.4% oxidation rate, were also noted [151]. Studies using ultrasound as a pre-treatment for the aqueous enzymatic oil extraction of thyme leaves (*Thymus vulgaris* L.) showed an increase in carvacrol, thymol, and p-cymene, while a significant decrease was observed for γ-terpinene [152]. Compounds such as E(Z)hexenal and nonanal were found in the volatiles from grapes (hybrid cultivar *Othello Vitis* sp.) extracted using ultrasound, and this was attributed to an enzymatic breakdown of unsaturated fatty acids during the injury-induced stress response to sample preparation [153].

## 4. Ultrasound-Assisted Production of Bio-Energy Products from Food Waste

### 4.1. Biodiesel Production

**Figure 6:**
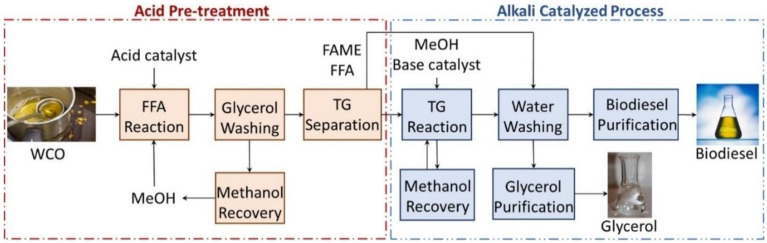
The direct transesterification of waste cooking oil (WCO) using acidic and alkaline catalysts [155]. WCO: waste cooking oil; FFA: free fatty acid; MeOH: methanol; TG: triglycerides; FAME: fatty acid methyl esters.

Biodiesel is sulphur-free, biodegradable, less toxic, and can be used in a compression ignition engine [154]. Biodiesel fuel is the alkyl esters of long-chain fatty acids. Biodiesel from food waste is obtained by the transesterification and alcoholysis of triacylglycerols such as waste fats, waste greases, and waste cooking oils [155]. There are two types of transesterifications: indirect and direct transesterification [156]. Normally, microorganisms can metabolize the carbon source into microbial oils for the indirect transesterification, whereas the alkaline or acidic catalysts in nature can be utilized for the direct transesterification (Figure 6). Ultrasound has been successfully applied in the transesterification reactions of waste cooking oils and the side-streaming products from oil production. Ultrasonic probe (horn) and bath have been employed in indirect/direct transesterification [155,157]. It has been recognized that the formation of emulsion between immiscible fluids generated by the mechanical effects was the major factor for increasing the direct/indirect transesterification reaction rate [158]. However, hydrogen and hydroxyl radicals have minor effects on the acceleration of this reaction [159]. Compared with high-frequency ultrasound, the mechanical effects are stronger in low-frequency ultrasound [160]. Therefore, low-frequency ultrasound is normally used for enhancing the biodiesel production from food waste. 

The ultrasound-assisted direct/indirect transesterification of food waste is summarised in Table 3. Carmona-Cabello et al. [161] reported that low-frequency ultrasound-assisted transesterification can significantly save both energy and reaction time to produce high-quality biodiesel from solid food waste. They also recommended the addition of an antioxidant in biodiesel to improve its storage stability. Oza et al. [162] optimised the methyl alcohol/oil molar ratio (6:1), temperature (50 °C), and the concentration of KOH (0.5%) for the ultrasound-assisted transesterification of waste cottonseed cooking oil; they found that the concentration of KOH was the major factor that influenced the yield of biodiesel. Similar results were found by Sharma et al. [163], who identified that the optimised methanol/oil molar proportion of ultrasound-assisted CaO and the KOH-catalysed transesterification of waste cottonseed cooking oil were 6.1:1 and 10.9:1, and that the biodiesel yield could reach 97.76% and 96.16%. In addition, the ultrasound-assisted conversion follows a pseudo-first-order reaction kinetics, and continuous sonication has higher effectiveness as compared with pulse sonication [163]. Yasvanthrajan et al. [164] found that the ultrasound-assisted transesterification could significantly shorten the reaction time (8 h reduction) and improve the biodiesel yield as compared with conventional processes. Meanwhile, the conversion of waste bio-oil was found to increase first, followed by a decrease with an increase in ultrasonic amplitude (from 20% to 100%) [164]. Apart from the above-mentioned catalysts, ultrasound has also been successfully employed in the biodiesel production from other catalysts, including modified coal fly ash [165], calcium diglyceroxide [166], and hydrotalcite [167].

### 4.2. Bio-Methane Production

Anaerobic digestion is the main pathway for producing biogas such as bio-methane and carbon dioxide from food waste [168]. Generally, the organic materials can be degraded by microorganisms in an anaerobic environment in order to generate bio-methane [169]. Due to the low yield of bio-methane during anaerobic digestion, ultrasound has been applied as a pre-treatment to solubilize the organic materials, disintegrate cell structure, and break the structure of cellulose crystalline so that the yield of bio-methane could be enhanced [170]. Some researchers have explored the effect of ultrasound on bio-methane production during anaerobic digestion (Table 4). Mirko et al. [171] compared the effect of five different pre-treatments (alkaline, thermo-alkaline, low temperature thermal, microwave, and ultrasound) on the yield of bio-methane during a 30-day anaerobic digestion. Compared with other pre-treatments, ultrasound has a minor influence on the biomethane yields. On the contrary, Yue et al. [17] reported that the bio-methane yield increased from 726.85 mL/g total volatile solids (TVS) to 927.97 mL/g TVS, with increased ultrasonic energy levels (from 1000 kJ/kg to 50,000 kJ/kg) during the 30-day anaerobic digestion of food waste. Shanthi et al. [172] found that the combination of surfactant addition and sonication pre-treatment could enhance the bio-methane during the 30-day anaerobic digestion of fruit and vegetable residue. Apart from the direct anaerobic digestion of food waste, it can also be co-digested with other materials such as manure, sludge, and crude glycerine [33]. Quiroga et al. [33] found that the daily production of bio-methane was increased by 31% for sonicated samples during the anaerobic co-digestion of food waste, cattle manure, and sludge. Similar results were also reported by Ormaechea et al. [173], in which the yield of bio-methane was increased by around two times in sonication pre-treated materials as compared with untreated materials after anaerobic co-digestion. Recently, researchers have tried to apply ultrasound in both the pre-treatment and the anaerobic processes. It was found that such a combination has a satisfactory influence on bio-methane production [40]. However, ultrasonic power, time, and duty cycle could affect such digestion progress and bio-methane production [40]. Therefore, the condition of ultrasound as a pre-treatment should be optimised according to the different substrates.

### 4.3. Bio-Hydrogen Production

Generally, food waste can be degraded into bio-methane and some other biogas such as bio-hydrogen from fermentation. Dark fermentation has been proven to be an effective pathway for producing bio-hydrogen [174]. Due to its mechanical effects, ultrasound can promote the production of bio-hydrogen by enhancing the solubilization of substrates. Up to now, ultrasound has been applied before and during the fermentation of food wastes (Table 5). Bundhoo et al. [60] compared the effects of ultrasound and microwave on dark fermentation from food and yard waste; they found that ultrasound was more effective at the solubilization of organic matter and contributed to a decrease in the production of bio-hydrogen. Elbeshbishy et al. [175] applied ultrasound to pre-treat food waste at 20 kHz and found that the longer pre-treatment time could significantly increase the yield of hydrogen. Later, their team compared the effects of four different pre-treatment methods, including ultrasound, heat shock, base, and acid, on the hydrogen yield [46]. In fact, ultrasonication pre-treatment reached the maximum production of hydrogen (894 mL), and such benefits could be inhibited by the combination of ultrasound with other processes, such as heat or base treatment [46]. Elbeshbishy et al. [56] investigated the effect of sonication on anaerobic hydrogen production from pulp waste and explained that sonication pre-treatment may promote the release of proteins and carbohydrates into the liquid phase to improve the yield of hydrogen (88% as compared to the control group). Similar results were also reported by Gadhe et al. [38], who found that the optimised ultrasonication conditions were 12 times and 8% solid content, and that this condition could enhance the yield of hydrogen by 75%. Additionally, Emmanuel et al. [176] also found that the hydrogen production of fermented palm oil mill effluent was increased by 38% after sonication pre-treatment, and that *Clostridium* spp. and *Thermoanaerobacterium* spp. were the dominant microflora in the fermented substrate. Arun and Sivashanmugam [49] also confirmed the positive effects of ultrasonication pre-treatment on dark fermentation. A 10 mL/g VS increase in the yield of hydrogen was observed in ultrasound pre-treated dairy waste as compared with untreated waste. However, apart from the enhancement of solubilization, other pathways still need to be explored.

### 4.4. Bio-Ethanol Production

Bio-ethanol is another product of food waste, and fermentation is the most common process to acquire this biogas. However, there is scant literature on the ultrasound-assisted production of bioethanol from food waste. Suresh et al. [177] reported that ultrasound pre-treatment at 340 W could improve the yield of bioethanol from potato waste after fermentation to 54.1 g/L. In addition, ultrasound-combined HCl was more efficient in the enhancement of bio-ethanol as compared with ultrasound-combined enzyme pre-treatment. Sindhu et al. [178] applied ultrasound-assisted alkali as a pre-treatment to ferment chili residue and obtained 1.94% bio-ethanol after fermentation. Battista et al. [179] compared different pre-treatment methods to improve bioethanol production from olive oil waste and found that ultrasound pre-treatment at 1800 W for 30 min could increase the yield of bio-ethanol by 50%. In addition, ultrasound was applied to hydrolyse sweet lime peel in a study on bio-ethanol production by John et al. [180], who confirmed that the maximum bio-ethanol yield after fermentation could reach 64%. Although the positive effects of ultrasound pre-treatment on bio-ethanol production have been proven, the mechanism, the effects of ultrasonic parameters, and the change in other properties during the fermentation of food waste are not clear and should be further investigated.

## 5. Conclusions and Perspectives

As discussed throughout the review, ultrasound has shown great potential as a green technology that can valorise various types of food waste materials in order to extract value-added products. Ultrasound accelerates the disintegration of large chunks of food waste into fine particles, thereby increasing the mass transfer and hence the diffusion of bioactives from food waste into the extraction medium. Ultrasound has been shown to rupture the cell walls of not only soft cells such as microalgae, but also hard cell walls such as plant cells. Ultrasound has been shown to be effective in extracting value-added materials from wheat waste, vegetable waste, oil palm fronds, wheat straw, rice straw, rice hull, sugarcane bagasse, and spent coffee waste. Ultrasonication can also be used to modify the crystal structure of the extracted materials. For plant cell walls, ultrasound has been proven to be a predominant approach for lignin barrier disruption, hemicellulose removal, and cellulose crystallinity reduction. Ultrasonication has proven to be an efficient method for extracting valuable materials from fruit and vegetable waste, oilseeds and nuts, pulse and legume processing waste, and cereal processing waste. From cereal processing waste, bioactive compounds such as phytosterols and β-glucans can be extracted by using ultrasonication.

From fruit and vegetable waste, ultrasonication increases the pectin output and reduces the extraction time. When ultrasonication was used, the pectin yields increased by 25% from the peels of pomegranate, orange, grapefruit, eggplant, grape pomace, and tomato waste. Ultrasonication can also reduce the concentration of unwanted components such as cyanide (HCN) and tannins, which are released into extraction medium.

In the algae biofuel industry, ultrasonication is proving to be an efficient method, not only for cell rupture to extract TAGs, but also for further transesterification reactions to convert the TAGs into biodiesel. In the case of enzymatic reactions, ultrasound enhances the reaction kinetics, thereby decreasing the time required and saving on costs. In the case of bio-methane production from anaerobic digestion, ultrasound has proven to be an efficient pre-treatment method for solubilizing the organic materials, disintegrating the cell structure, and breaking the structure of cellulose crystalline so that the yield of bio-methane could be enhanced. In the case of bio-hydrogen production from the fermentation of food waste, ultrasound can promote the production of bio-hydrogen by enhancing the solubilization of substrates. Ultrasound was shown to be effective in producing bio-hydrogen before and during the fermentation of food wastes. In the case of bioethanol production from the fermentation of potato waste, ultrasonication has been shown to increase the bioethanol yield.

Although ultrasonication has proven to be effective in extracting valuable materials from food and agriculture wastes, more research needs to be performed on the cost-effectiveness of the treatment when applied in a large scale. There have been a lot of developments in the scaling up of ultrasonic extraction technologies. A number of companies are producing flow-through sonication systems to reduce the footprint of ultrasonic reactors. The flow-through systems work well when the viscosities of the sonication medium are low to medium. However, when the viscosities increase during the sonication, the effectiveness may decrease with time. There are few studies in the literature that have studied the optimum viscosity levels of algae slurry so that cells could be ruptured well and oils could be extracted efficiently [14,181]. The energy input into the system for the unit extraction of value-added materials was calculated and compared with existing technologies. These kinds of in-depth studies are needed so that the energy efficiency of ultrasonication can be proved.

## Figures and Tables

**Table 1:** The influence of ultrasound on SCOD changes.

Source/Composition of Food Waste	Ultrasonic Conditions	Outcomes	Reference
A mixture of rice, cabbage, pork, and tofu waste with different total solid (TS) content	20 kHz, 480 W/L, 15 min	TS = 40 g/L, ΔSCOD: +157% TS = 100 g/L, ΔSCOD: +172%	[39]
Sewage sludge	27 kHz, 200 W/L	2.5 min, ΔSCOD: +239% 10 min, ΔSCOD: +577%	[41]
Activated sludge	20 kHz, 21 kJ/g TS, 9 min	ΔSCOD: +246%	[47]
A mixture of wheat, gram flour, rice, fruit peel, and vegetable waste	20 kHz, 0.4 W/mL, 10 min	ΔSCOD: +61.5%	[40]
Food waste obtained from the Dufferin Organics Processing Facility	20 kHz, 79 kJ/g TS	ΔSCOD: +25%	[46]
Food waste from the Aurora treatment plant	20 kHz, 10,384, 15,577, 20,769 kJ/kg TS for 30, 45, 60 min US sample	30 min, ΔSCOD: +10.3% 45 min, ΔSCOD: +29.4% 60 min, ΔSCOD: +37%	[48]
Dairy waste	20 kHz, 0.6 W/mL, 25 min	ΔSCOD: +28.4%	[49]
Complex food waste	20 kHz, 16,875 kJ/kg TS, 15 min	ΔSCOD: +56.5%	[38]
Algae	20 kHz, 30 min, 36,000 KJ/Kg TS	Maximum ΔSCOD: +1950% was observed at 200 W US power	[50]
Pulp mill	20 kHz, 3.1 W/mL	15 min, ΔSCOD: +14.9% 60 min, ΔSCOD: +44.3%	[42]
Food waste	20 kHz, 5000 kJ/kg	ΔSCOD: +9.0%	[51]
Rice, noodles, vegetables, and meat waste obtained from a cafeteria at Harbin Institute	20 kHz, 1.25 W/mL, 30 min	ΔSCOD: +115%	[52]
Activated sludge	24 kHz, 1690 and 3380 kJ/kg TS for 5- and 10 min sonication sample	5 min, ΔSCOD: +17% 10 min, ΔSCOD: +21%	[43]
Activated sludge	20 kHz, 1.04 W/mL, 2.5 min	ΔSCOD: +12.6%	[53]
Food waste	20 kHz, 2 W/mL, 15 min	ΔSCOD: +71.5%	[54]
Algae sludge	40 kHz, 30 min, the power density was not mentioned	ΔSCOD: +520%	[55]
Food waste	20 kHz, 23 kJ/g TS, 30 min	ΔSCOD: +22.1%	[56]
Digestate	20 kHz, 3000 kJ/kg TS	ΔSCOD: +21%	[57]
Organic waste from the food industry	20 kHz, 50,000 kJ/kg TS	ΔSCOD: +20%	[58]
Dairy digestate waste	20 kHz, 15,000 kJ/kg TS	ΔSCOD: +15%	[59]
Food waste	20 kHz, 6946 kJ/kg TS, 30 min	ΔSCOD: +159%	[60]
Solid organic waste	20 kHz, 15,000 kJ/kg TS	ΔSCOD: +9.0%	[61]
Olive mill wastewater	20 kHz, 0.4 W/mL, 10 min	ΔSCOD: +23%	[62]
Solid waste	20 kHz, 0.2 W/mL, 60 min	ΔSCOD: +18.5%	[63]
Fermentation residues	20 kHz pulsed US (4 s on, 6 s off), 2 W/mL, 30 min	ΔSCOD: +39.5%	[25]
Seed sludge from food factory	20 kHz, 200 W/L, 45 min	ΔSCOD: +11%	[64]

**Table 2:** The variation in the amounts of cellulose, hemicellulose, and lignin in an extract after ultrasonic treatment.

Source/Composition of Food Waste	Ultrasonic Conditions	Outcomes	Reference
Wheat waste	20 kHz, 30 min, NaOH (2% *w*/*v*) combination	Δcellulose: +13.2%; Δlignin: −10.1%	[88]
Vegetable waste	20 kHz, 20 min	Δcellulose: +23.1%; Δhemicellulose: −9.0%; Δlignin: −10.2%	[82]
Oil palm fronds	20 kHz, 50 min	Δlignin: −5.8%	[89]
Wheat straw	20 kHz, 120 min at 50 °C	Δlignin: −6.2%	[90]
Rice straw	30 kHz, water bath 90 °C, 4 h	Δlignin: −4.6%	[92]
Rice hull	40 kHz, 500 W, 1.5 h	Δcellulose: −2.8%; Δhemicellulose: −3.7%; Δlignin: +1.6%	[86]
Sugarcane bagasse	22 kHz, 50 W, 25 min, ozone/alkaline assisted	Δcellulose: +10.5%; Δhemicellulose: −8.1%; Δlignin: −15.6%	[83]
Sugarcane bagasse	24 kHz, 500 W, water bath 40 °C	Δcellulose: +21.4%; Δhemicellulose: −18.6%; Δlignin: −8.4%	[84]
Spent coffee waste	47 kHz, 310 W, 20 min	Δlignin: −8.8%	[93]

**Table 3:** Ultrasound-assisted transesterification of food waste to produce biodiesel.

Materials	Ultrasonic Frequency	Ultrasonic Power	Temperature	Biodiesel Yield	Reference
Solid food waste oil	20 kHz	50% amplitude	52.5 °C	93.23%	[161]
Waste cottonseed cooking oil	20 kHz	500 W	50 °C	98%	[162]
Waste cottonseed cooking oil	20 kHz	500 W	40–60 °C	70.21~97.76%	[163]
Waste bio-oil	20 kHz	20~100% amplitude	45 °C	98.7%	[164]
Waste cooking oil	20 kHz	108 W	70 °C	95.57%	[165]
Waste cooking oil	22 kHz	120 W	60 °C	93.5%	[166]
Waste cooking oil	20 kHz	55% amplitude	57 °C	76.45%	[167]

**Table 4:** Effect of ultrasound on bio-methane production from food waste during anaerobic digestion.

Materials	Ultrasonic Frequency	Ultrasonic Power	Temperature	Bio-Methane Yield	Reference
Buckwheat hull	40 kHz	110 W	25 °C	141.9 NL Kg VS-1	[171]
Restaurant waste	20 kHz	0–500 W	Room temperature	647.49 mL/g TVS~927.97 mL/g	[17]
Fruit and vegetable residue	25 kHz	90 W	-	0.61 g/g COD	[172]
Mixture of food waste, cattle manure, and sludge	24 kHz	400 W	-	0.85 L/L day	[33]
Mixture of food waste, cattle manure, and crude glycerine	24 kHz	400 W	55 °C	520 L/kg VS	[173]
Food waste	20 kHz and 25 kHz	120 W and 200 W	35 °C	~0.26 L/day	[40]

**Table 5:** Effect of ultrasound on bio-hydrogen production from food waste during fermentation.

Materials	Ultrasonic Frequency	Ultrasonic Power	Temperature	Bio-Hydrogen Yield	Reference
Mixture of food and yard waste	20 kHz	0~500 W	-	7.87 mL/g VS, decreased by 11%	[60]
Food waste	20 kHz	500 W	<30 °C	1.55 mol/mol VS, increased by 120%	[175]
Food waste	20 kHz	500 W	<30 °C	97 mL/g VS, increased by 131%	[46]
Food waste	20 kHz	500 W	<30 °C	80 mL/g VS, increased by 88%	[56]
Food waste	20 kHz	1200 W	<30 °C	0.62~5.23 mL/h, increased by 75%	[38]
Food waste	20 kHz	130 W	-	0.7 mmol/g COD, increased by 38%	[176]
Dairy waste	20 kHz	0.3~1.1 W/mL	25 °C	15.51 mL/g VS, increased by 3 times	[49]

## Data Availability

Not applicable.
